# Kidney organoids reveal redundancy in viral entry pathways during ACE2-dependent SARS-CoV-2 infection

**DOI:** 10.1128/jvi.01802-23

**Published:** 2024-02-09

**Authors:** Jessica M. Vanslambrouck, Jessica A. Neil, Rajeev Rudraraju, Sophia Mah, Ker Sin Tan, Ella Groenewegen, Thomas A. Forbes, Katerina Karavendzas, David A. Elliott, Enzo R. Porrello, Kanta Subbarao, Melissa H. Little

**Affiliations:** 1 The Novo Nordisk Foundation Centre for Stem Cell Medicine (reNEW), Murdoch Children’s Research Institute, Melbourne, Australia; 2 Department of Paediatrics, Faculty of Medicine, Dentistry and Health Sciences, The University of Melbourne, Melbourne, Australia; 3 Department of Microbiology and Immunology, The Peter Doherty Institute for Infection and Immunity, The University of Melbourne, Melbourne, Australia; 4 Department of Nephrology, Royal Children’s Hospital, Melbourne, Australia; 5 Australia Regenerative Medicine Institute, Monash University, Melbourne, Victoria, Australia; 6 Melbourne Centre for Cardiovascular Genomics and Regenerative Medicine, The Royal Children’s Hospital, Melbourne, Australia; 7 Department of Anatomy and Physiology, School of Biomedical Sciences, The University of Melbourne, Melbourne, Australia; 8 The WHO Collaborating Centre for Reference and Research on Influenza, The Peter Doherty Institute for Infection and Immunity, Melbourne, Australia; 9 Novo Nordisk Foundation Centre for Stem Cell Medicine (reNEW), Faculty of Health and Medical Sciences, University of Copenhagen, Copenhagen, Denmark; The Ohio State University, Columbus, Ohio, USA

**Keywords:** SARS-CoV-2, COVID-19, kidney, kidney organoids, stem cells

## Abstract

With a high incidence of acute kidney injury among hospitalized COVID-19 patients, considerable attention has been focussed on whether SARS-CoV-2 specifically targets kidney cells to directly impact renal function, or whether renal damage is primarily an indirect outcome. To date, several studies have utilized kidney organoids to understand the pathogenesis of COVID-19, revealing the ability for SARS-CoV-2 to predominantly infect cells of the proximal tubule (PT), with reduced infectivity following administration of soluble ACE2. However, the immaturity of standard human kidney organoids represents a significant hurdle, leaving the preferred SARS-CoV-2 processing pathway, existence of alternate viral receptors, and the effect of common hypertensive medications on the expression of ACE2 in the context of SARS-CoV-2 exposure incompletely understood. Utilizing a novel kidney organoid model with enhanced PT maturity, genetic- and drug-mediated inhibition of viral entry and processing factors confirmed the requirement for ACE2 for SARS-CoV-2 entry but showed that the virus can utilize dual viral spike protein processing pathways downstream of ACE2 receptor binding. These include TMPRSS- and CTSL/CTSB-mediated non-endosomal and endocytic pathways, with TMPRSS10 likely playing a more significant role in the non-endosomal pathway in renal cells than TMPRSS2. Finally, treatment with the antihypertensive ACE inhibitor, lisinopril, showed negligible impact on receptor expression or susceptibility of renal cells to infection. This study represents the first in-depth characterization of viral entry in stem cell-derived human kidney organoids with enhanced PTs, providing deeper insight into the renal implications of the ongoing COVID-19 pandemic.

**IMPORTANCE:**

Utilizing a human iPSC-derived kidney organoid model with improved proximal tubule (PT) maturity, we identified the mechanism of SARS-CoV-2 entry in renal cells, confirming ACE2 as the sole receptor and revealing redundancy in downstream cell surface TMPRSS- and endocytic Cathepsin-mediated pathways. In addition, these data address the implications of SARS-CoV-2 exposure in the setting of the commonly prescribed ACE-inhibitor, lisinopril, confirming its negligible impact on infection of kidney cells. Taken together, these results provide valuable insight into the mechanism of viral infection in the human kidney.

## INTRODUCTION

With ongoing morbidity, mortality, and emergence of new SARS-CoV-2 variants, COVID-19 continues to be a significant health concern worldwide (1). Acute kidney injury (AKI) has been estimated to affect 20%–25% of hospitalized COVID-19 patients [reviewed in references (2, 3)], leading to an increased case fatality rate [>60% (4–6)] and risk of chronic kidney disease (CKD) following discharge regardless of their previous renal condition (7, 8). The specific link between SARS-CoV-2 infection and AKI remains incompletely understood. While renal damage has been reported to arise from indirect factors, such as complement system activation and inflammatory responses [reviewed in references (2, 9)], with conflicting reports of SARS-CoV-2 renal tropism (10–12), clear lines of evidence have suggested a direct mechanism of kidney injury. In addition to established high rates of AKI in hospitalized patients, numerous studies have reported evidence of direct kidney cell infection within postmortem samples, frequently alongside AKI (13–17). Such findings have been strengthened by the detection of renal cell infection *in vitro* (15, 18–22) in addition to biomarkers of tissue injury (15, 21, 23–25).

Despite this strong evidence, kidney damage following COVID-19 is speculated to be multifactorial in nature, potentially involving direct renal cell infection, organ crosstalk, inflammation, and genetic factors including viral entry factor expression (2, 26). SARS-CoV-2 entry into cells requires binding of the viral spike (S) protein and the cell receptor (ACE2) followed by proteolytic cleavage of the S protein, driving S protein activation and fusion of the viral membrane with the cell [reviewed in reference (27)]. These critical viral entry steps can occur via two different pathways: (i) entry utilizing cell surface/plasma membrane-associated protease of the transmembrane serine protease (TMPRSS) family or (ii) entry utilizing endosomal-associated proteases of the Cathepsin family. The requirement for both the ACE2 receptor and the protease TMPRSS2 in facilitating host cell entry of SARS-CoV-2 has now been well established in multiple tissues (27–30). The human kidney is composed of millions of tubular blood filtration units known as nephrons that are structurally and functionally segmented along their length. The earlier portion of the nephron, known as the PT, expresses high levels of ACE2 (17, 28, 31–33). In contrast, TMPRSS2 expression is predominantly localized to the later portion of the nephron (distal tubules). This incomplete overlap of ACE2 and TMPRSS2 expression suggests the possibility of alternate entry mechanisms (21, 23, 26, 34–36), with this prospect strengthened by the description of several alternate receptors and proteases across a range of tissues (37–41).

Further complicating COVID-19 disease risk and outcome is the frequent presence of comorbidities. Pre-existing CKD represents the most common comorbidity in COVID-19 patients and is associated with a poor prognosis [reviewed in references (2, 3, 42–44)]. However, the large proportion of CKD patients taking renin-angiotensin system (RAS) inhibitors has revealed deeper complexities with respect to patient risk and management (45). While it is now generally accepted that patients should not discontinue prescribed RAS inhibitors [reviewed in references (2, 46)], there have been conflicting reports of the influence of these drugs on ACE2 expression (46–49) and whether this is sufficient to alter host cell entry or increase tissue infection.

Stem cell-derived human kidney organoids have previously been exploited to understand COVID-19 pathogenesis (15, 18–22). These studies indicated that SARS-CoV-2 preferentially, but not exclusively, infects PTs and can cause cellular injury, while reduced infection was observed following organoid exposure to soluble ACE2 (15, 18–22). However, the existence of alternate viral receptors, the entry pathway utilized downstream of ACE2 binding for viral spike protein priming, and the role of ACE2 inhibitors on infection are unknown or poorly understood. A key challenge in this field has been the immaturity of PT segments within kidney organoid PTs since it is the PTs that are the primary targets of SAS-CoV-2 in the kidney (21, 50). In the current study, we sought to overcome this challenge by using our previously reported stem cell-derived PT-enhanced organoid model which is produced by differentiating pluripotent stem cells to nephron progenitors in a manner that better recapitulates the precise timing and signaling environment of human kidney development. The resulting organoids possess improved PT maturity, more suited to investigating the mechanisms of SARS-CoV-2 infection of human kidney (21). Here, our studies utilizing PT-enhanced organoids revealed that, while ACE2 represents the sole SARS-CoV-2 receptor in renal cells, redundancy exists in the utilization of Cathepsin L (CTSL)- and TMPRSS-mediated endocytic and non-endosomal viral processing pathways. We also demonstrate that viral entry factor expression and organoid infectivity are not increased in the presence of the ACE-inhibitor (ACEi), lisinopril, suggesting that these medications do not heighten the risk of direct renal cell infection.

## RESULTS

### PT-enhanced organoids show extensive viral entry factor expression

**Fig 1:**
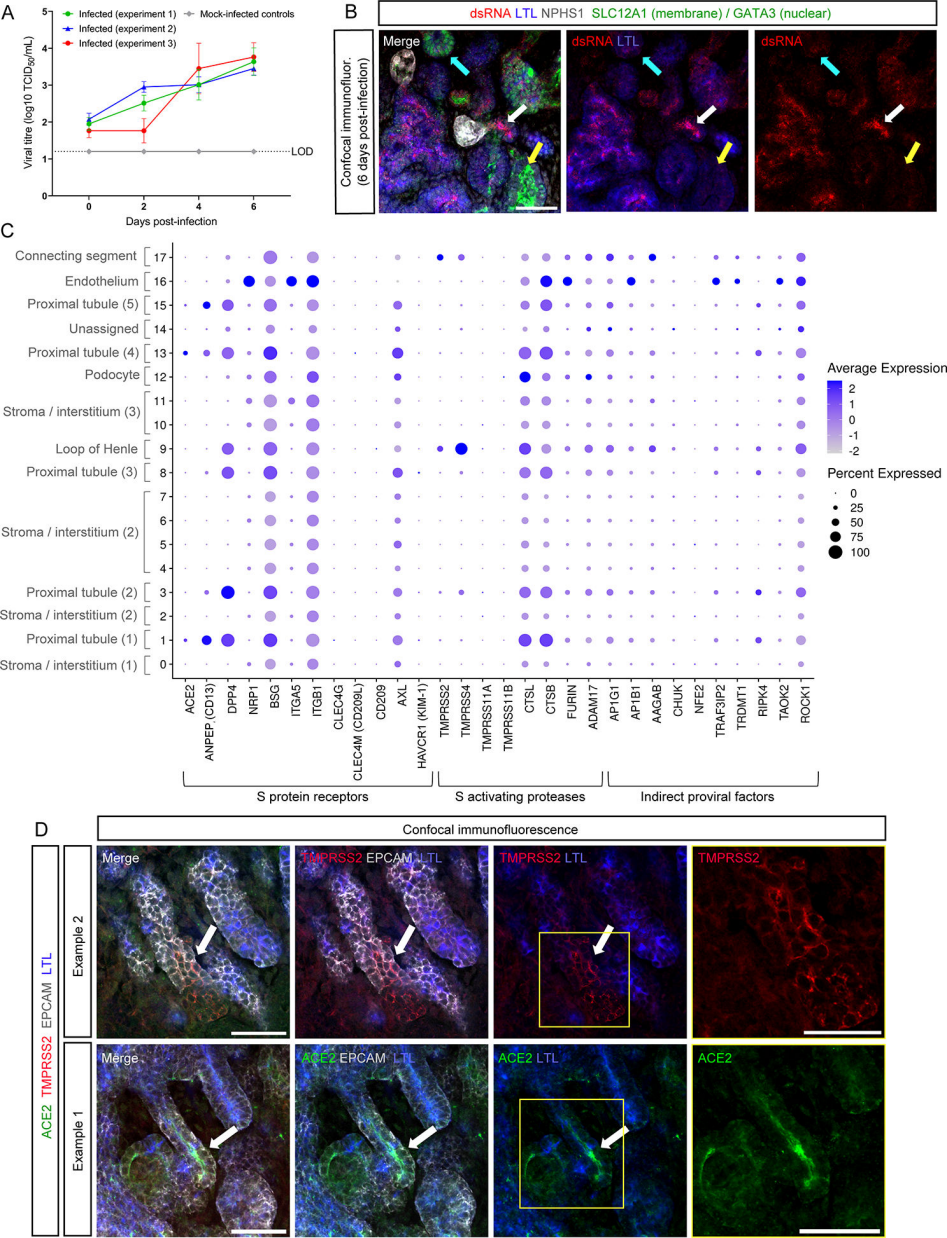
PT-enhanced organoids show extensive viral entry factor expression. (**A**) Line plot depicting viral titre as determined by Vero cell assays (Median Tissue Culture Infectious Dose; log_10_ TCID_50_) of culture media sampled from WA1 SARS-CoV-2 (icSARS-CoV-2-GFP)-infected PT-enhanced organoids across three independent experiments replicated identically (red, green, and blue lines), as well as mock-infected organoids (gray line). Mock-infected line is representative of all mock results across each experiment. LOD and dotted line represent lower limit of detection. Error bars represent standard error of the mean (SEM) from *n* = 3 individual wells of organoids (three organoids per well) at each timepoint (note these data represent the “no drug controls” from experiments depicted in Fig. 3A). (**B**) Confocal immunofluorescence of a representative PT-enhanced organoid 6 days post-infection, demonstrating SARS-CoV-2 double stranded RNA (dsRNA; red) localization, co-stained for markers of proximal tubules (LTL; blue), loop of Henle (SLC12A1; green, apical membrane staining), late distal tubule/connecting segment (GATA3; green, apical), and podocytes of the glomeruli (NPHS1; gray). Arrows indicate examples of a LTL-positive tubule (white arrow), SLC12A1-positive tubule (yellow arrow), and GATA3-positive tubule (cyan arrow). Scale bar represents 50 µm. (**C**) scRNAseq DotPlot of PT-enhanced organoids (day 14 of organoid culture) depicting the expression of entry factors (receptors and proteases) and pro-viral factors reported in literature to be involved in SARS-CoV-2 infectivity. Identities of each cluster are labeled (left axis), with numbers in brackets differentiating kidney cell populations for which multiple clusters exist. (**D**) Confocal immunofluorescence of PT-enhanced organoid examples depicting ACE2 expression (green) within LTL + proximal tubules (blue) and TMPRSS2 (red) in LTL structures. Epithelium is marked with EPCAM (gray). Arrows depict examples of ACE2 and TMPRSS2 staining. Yellow squares highlight fields of view shown at higher magnification in the images on far right. Scale bars represent 50 µm.

To confirm that previous reports of SARS-CoV-2 infection predominantly targeting PT segments in human kidney tissue (17, 25) and standard kidney organoids (15, 18–22) were similarily reproducible in the PT-enhanced organoid model, PT-enhanced organoids were infected with 10^4^ tissue-culture infectious dose (TCID) of ancestral SARS-CoV-2 (WA1) at day 14 of organoid culture (Fig. S1A). Measurement of virus levels in the organoid culture media from days 0–6 post-infection revealed increased levels of infectious virus as early as day 2 (Fig. 1A). These findings were supported by immunofluorescence of organoids 6 days post-infection by co-staining for double-stranded RNA (dsRNA) and markers of kidney structures (Fig. 1B). In agreement with reports of SARS-CoV-2 tropism for PTs (15, 17, 19, 21, 25, 51, 52), virus was predominantly detected in PT segments [marked here with *Lotus tetragonobulus* lectin (LTL)], confirming PTs as a major target for infection and further validating the suitability of the enhanced organoid model for subsequent experiments (Fig. 1B).

Expression levels and cellular distributions of SARS-CoV-2 entry factors have not been well described in the context of kidney organoids. Furthermore, the observation that ACE2 and TMPRSS2 are expressed on distinct renal cell types suggests that the virus may utilize alternate combinations of receptors and proteases (23). To investigate further, a single cell RNA sequencing (scRNAseq) data set from our PT-enhanced organoids (21) was analyzed for a broad range of entry factors curated from literature, including S protein receptors, proteases, and proviral factors (thought to indirectly increase infectivity) (28, 37, 39, 40, 53–62) (Fig. 1C). Overall, the expression of viral entry and proviral factors were more abundant in PT clusters (1, 3, 8, 13, and 15), correlating with their permissiveness to infection (Fig. 1C). While *ACE2*/ACE2 gene and protein expression were confirmed in PT cells, *TMPRSS2*/TMPRSS2 displayed a predominantly distal nephron localization, with the highest gene expression in connecting segment/loop of Henle and protein expression in LTL-negative (distal) nephron epithelium, consistent with previous findings (Fig. 1CD) (23, 26, 34–36, 63). The distribution of S protein receptors and S activating proteases resembled previously published expression patterns in human fetal kidney scRNAseq data sets (21). In particular, *CSTB* and *CSTL* were highly expressed in several cell clusters, including the PT clusters (Fig. 1C). Overall, these data suggested that SARS-CoV-2 infection of PT segments may be independent of TMPRSS2.

### Improved stem cell-derived PT confirms a sole receptor for SARS-CoV-2 entry in renal cells

The role of ACE2 in viral entry has been established in a range of tissues, including standard kidney organoids (15, 18–22). While standard kidney organoids showed evidence of significantly reduced viral RNA and viral titres following ACE2 neutralization and (19, 20, 22) KO, incomplete ablation has lent support to reports of alternate receptors in renal cells (56, 64). Given their improved PT maturity and, thus, human-relevance, PT-enhanced organoids were utilized to assess the dependency of SARS-CoV-2 entry on ACE2 expression.

**Fig 2:**
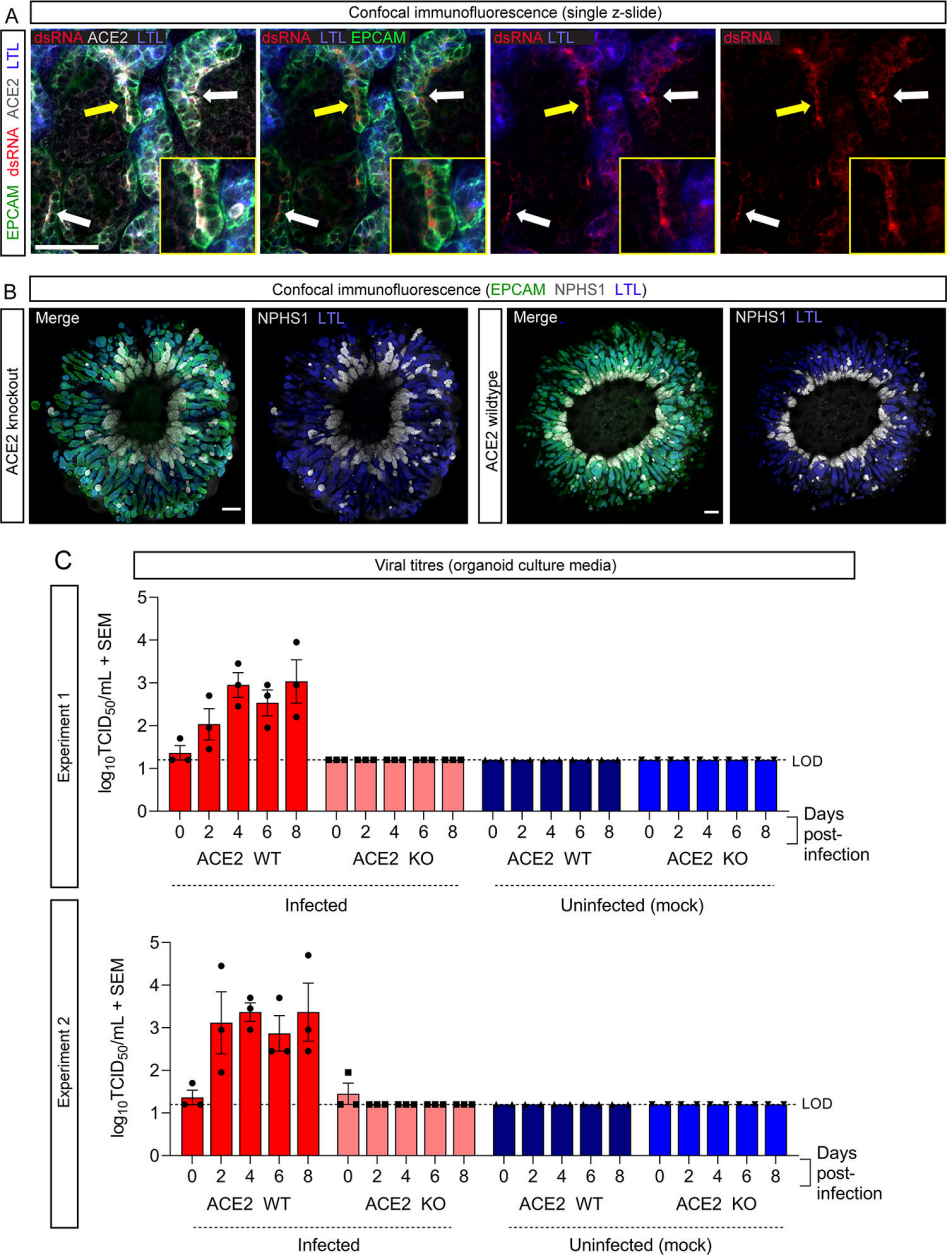
ACE2 is the sole receptor for SARS-CoV-2 in renal cells. (**A**) Confocal immunofluorescence of a representative PT-enhanced organoid 6 days post-infection demonstrating SARS-CoV-2 double-stranded RNA (dsRNA; red) localization within ACE2-positive (gray) proximal tubule cells. Organoid is co-stained for markers of proximal tubule brush-border membrane (LTL; blue) and nephron epithelium (EPCAM; green). Arrows indicate examples of dsRNA staining, with yellow arrow indicating the region shown at higher magnification in the inset images. Scale bar represents 50 µm. (**B**) Confocal immunofluorescence of PT-enhanced organoids (day 14 of organoid culture) generated from ACE2 knockout and wild-type iPSCs, depicting nephron epithelium (EPCAM; green), podocytes of the glomeruli (NPHS1; gray), and proximal tubules (LTL; blue). Scale bars represent 200 µm. Each confocal image depicts 3 × 3 stitched tiles, generated using the standard rectangular grid tile scan mode with automated stitching during image acquisition using ZEISS ZEN Black software (Zeiss Microscopy, Thornwood, NY) installed on a ZEISS LSM 780 confocal microscope (Carl Zeiss, Oberkochen, Germany). (**C**) Bar graphs from two independent experiments (top and bottom) depicting the viral titres (log_10_ TCID_50_/mL) of culture media sampled from ACE2 knockout (KO) and wild-type (WT) PT-enhanced organoids, both infected with VIC01 SARS-CoV-2 (dark and light red bars) or remaining uninfected (controls; light and dark blue bars). Error bars represent SEM from three biological replicates per timepoint. LOD, lower limit of detection.

PT-enhanced kidney organoids were first assessed for a similar dependence on ACE2 for viral entry as suggested in previous studies utilizing standard organoids. Immunofluorescence of enhanced organoids for dsRNA confirmed viral RNA co-localization with LTL-positive PTs co-expressing the ACE2 receptor (Fig. 2A), supporting the role of ACE2 in viral entry in this model. Organoids were then generated from published *ACE2* knockout (ACE2 KO) and wild-type control (ACE2 WT) iPSC lines (parental line: MCRIi010-A, peripheral blood mononuclear cell-derived iPSCs), generated and characterized previously (29). Both iPSC lines generated organoids with the expected morphology and patterning of PT-enhanced organoids, with radially aligned and proximalized nephrons (Fig. 2B) (21).

To investigate the role of ACE2, organoids were infected with 10^4^ TCID of VIC01SARS-CoV-2 and the culture media sampled up to day 6 post-infection. Infectious virus was detected in ACE2 WT cultures by day 2 post-infection across independent experiments replicated using identical conditions (Fig. 2C). In contrast, no virus was detected in ACE2 KO cultures. These findings confirm that ACE2 receptor expression is critical for SARS-CoV-2 infection of PTs. Furthermore, the complete ablation of infectivity upon ACE2 KO using this improved model of the human PT strongly suggests that human renal cells *in vivo* do not possess an alternate SARS-CoV-2 receptor.

### SARS-CoV-2 utilizes dual viral entry pathways in renal cells

**Fig 3:**
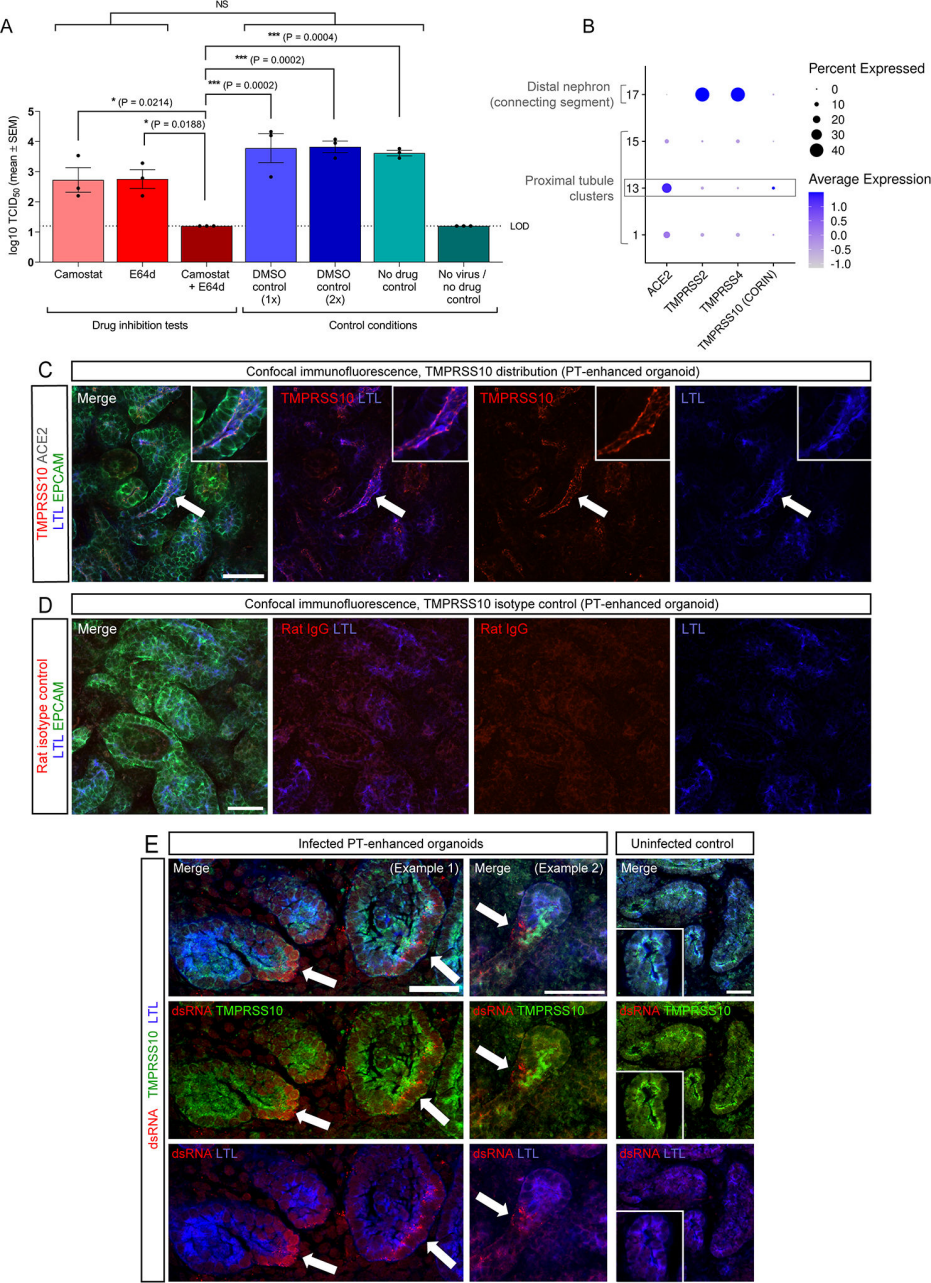
Drug inhibition assays revealing dual viral entry pathway usage by SARS-CoV-2. (**A**) Bar graph depicting the viral titres (log_10_ TCID_50_/mL) of culture media sampled from PT-enhanced organoids 6 days post-infection, treated with either protease inhibitors (Camostat and E64d, alone or in combination; dark/light red bars), drug reconstitution reagent (DMSO controls; dark/light blue bars), or remaining untreated (no drug controls; light/dark green bars). PT-enhanced organoids were infected with WA1 SARS-CoV-2. (icSARS-CoV-2-GFP). LOD and dotted line represent lower limit of detection. Error bars represent SEM from three independent experiment, with three (drug inhibition tests and no drug controls) or two (DMSO controls) biological replicates per timepoint. Significance was calculated using a one-way ANOVA with Tukey’s multiple comparisons test. Asterisks represent two-tailed *P* values (*P ≤ 0.05, **P ≤ 0.01, ***P ≤ 0.001, ****P ≤ 0.0001). NS, not significant. (**B**) scRNAseq DotPlot of PT-enhanced organoids (day 14 of organoid culture) depicting the expression of *TMPRSS* genes within the highest *ACE2*-expressing proximal tubule clusters and distal (connecting segment) nephron cluster. The proximal tubule cluster most enriched with *ACE2* expression is outlined in gray. Dot size represents the percentage of cells expressing a gene within each cluster, while shade intensity correlates with gene expression level. (**C and D**) Confocal immunofluorescence of TMPRSS10 (C, red) and corresponding rat isotype control (D, red), co-stained with markers of proximal tubule (LTL, blue) and nephron epithelium (EPCAM, green). Arrows indicate region shown at higher magnification in inset images. Scale bars represent 50 µm. (**E**) Confocal immunofluorescence of SARS-CoV-2-infected and uninfected (control) PT-enhanced organoids, depicting virus (dsRNA, red) within TMPRSS10-expressing (green) proximal tubules (marked with LTL, blue) within infected samples and lack of dsRNA detection in uninfected control. Arrows depict examples of dsRNA staining. Scale bars represent 50 µm.

Having confirmed SARS-CoV-2 entry is dependent on ACE2 using PT-enhanced organoids, the downstream mechanisms of viral entry in renal cells were interrogated by pre-treating PT-enhanced organoids with drug inhibitors of the TMPRSS family (Camostat mesylate) (65) and Cathepsin (CTSL and CTSB: E64d/Aloxistatin) proteases (41, 66). Viral titres confirmed successful infection of organoids treated with Camostat alone, E64d alone, and DMSO (drug reconstitution reagent control) (Fig. 3A; Fig. S1B). Interestingly, a 10-fold reduction in viral titre was observed following treatment of organoids with each of the individual inhibitors compared to DMSO-treated controls although this did not reach statistical significance (Fig. 3A). In contrast, combined treatment (Camostat + E64 d) consistently prevented PT-enhanced organoid infection, together suggesting utilization of both entry pathways by SARS-CoV-2.

In agreement with drug inhibition assay results, *CTSL* and *CTSB* were highly expressed in the majority of cells in PT clusters 1, 13, and 15 [“Proximal tubule (1),” “Proximal tubule (4),” and “Proximal tubule (5)”] (Fig. S3C). Owing to the low expression of *TMPRSS2* in *ACE2*-expressing PT clusters (Fig. 1C), PT-enhanced organoids were re-analyzed for a broader range of *TMPRSS* family genes thought to play roles in SARS-CoV-2 infection and similarly inhibited by Camostat (40) (Fig. 3B; Fig. S1C). In addition to *TMPRSS2*, *TMPRSS4* and *TMPRSS10* expression levels in the organoids were also of note (Fig. S1C). However, the PT cluster displaying highest *ACE2* expression [cluster 13: “Proximal tubule (4)”] primally expressed *TMPRSS10*, with *TMPRSS2* and *TMPRSS4* being largely restricted to distal nephron clusters (e.g., loop of Henle, connecting segment) (Fig. 3B). These results suggested that the reduced infection in Camostat-treated organoids could be attributed to *TMPRSS10* inhibition and was supported by protein localization studies (Fig. 3C through E; Fig. S1D and E). TMPRSS10 protein expression was observed on the apical membrane of LTL+/EpCAM + PTs within enhanced kidney organoids (Fig. 3C and D), with the presence of viral RNA (dsRNA) within TMPRSS10-expressing PTs confirmed at 6 days post-infection (Fig. 3E). In contrast, TMPRSS4 was not detectable above background levels (Fig. S1D and E).

Taken together, these results supported a capacity for SARS-CoV-2 to utilize both TMPRSS- and CTSL/CTSB-mediated pathways and suggested a possible role for TMPRSS10 in renal cells.

### ACEi lisinopril does not increase susceptibility of renal cells to SARS-CoV-2

**Fig 4:**
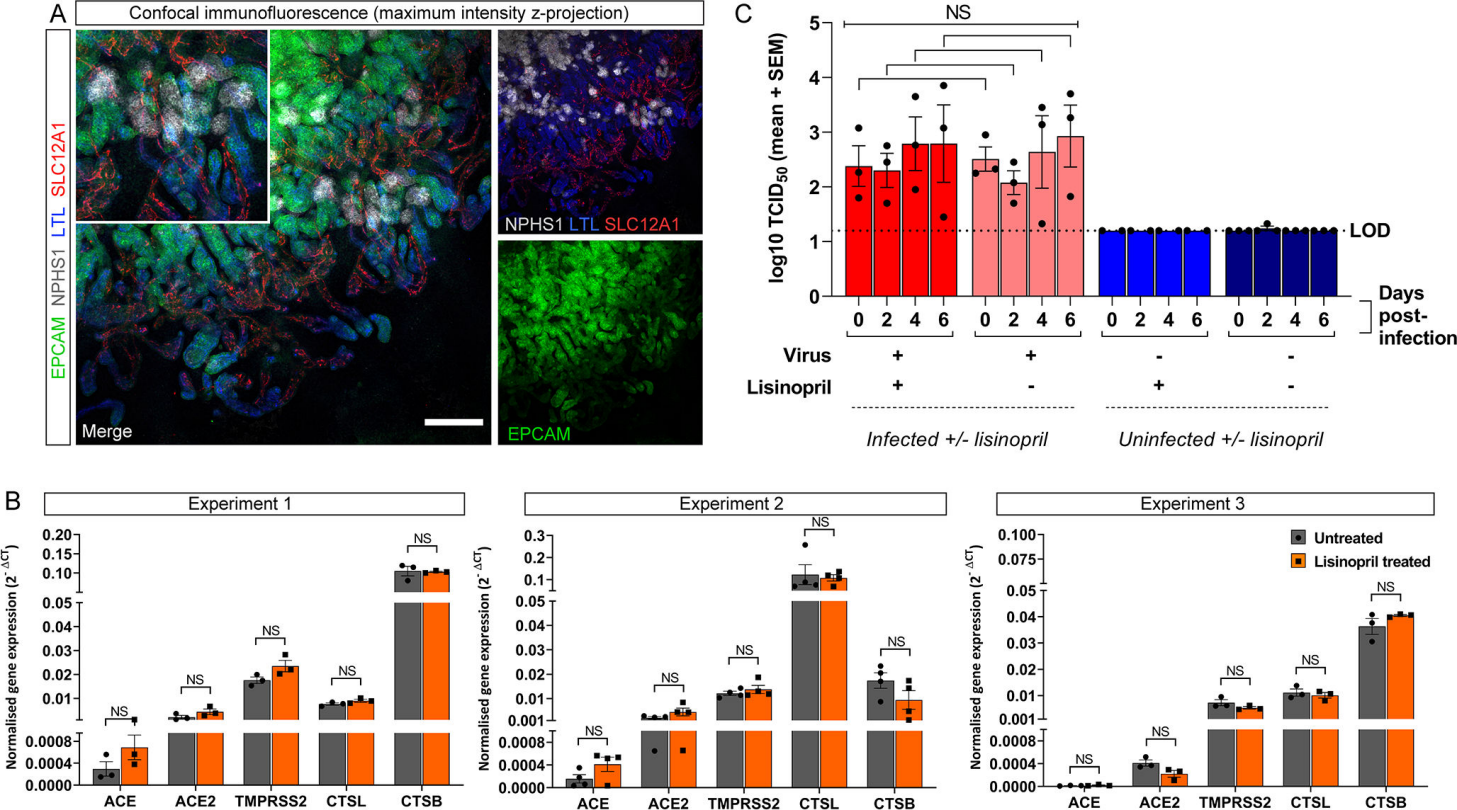
PT-enhanced organoids do not show increased susceptibility to SARS-CoV-2 infection following lisinopril exposure. (**A**) Confocal immunofluorescence of a representative PT-enhanced organoid following 8 days of lisinopril treatment, depicting nephron epithelium (EPCAM; green), podocytes of the glomeruli (NPHS1; gray), proximal tubules (LTL; blue), and loop of Henle (SLC12A1; red). Inset depicts a region of the same merge image shown at higher magnification. Scale bar represents 200 µm. (**B**) qRT-PCR analyses of untreated (gray bars) and lisinopril-treated (orange bars) PT-enhanced organoids (14 days of organoid culture) from three independent experiments for expression of the lisinopril target, *ACE,* and relevant SARS-CoV-2 entry factors. Error bars represent SEM from three biological replicates. Statistical significance was assessed using an unpaired *t* test. NS, not significant. (**C**) Bar graph depicting the viral titres (log_10_ TCID_50_/mL) of culture media sampled from untreated and lisinopril-treated PT-enhanced organoids, either infected with WA1 SARS-CoV-2 (icSARS-CoV-2-GFP) 48 h post-treatment (light/dark red bars) or remaining uninfected (light/dark blue bars). LOD and dotted line represent lower limit of detection. Significance was assessed using unpaired *t* tests adjusted for multiple comparisons using the Holm-Sidak method. NS, not significant.

To investigate the potential effect of ACEi on renal infectivity to SARS-CoV-2, PT-enhanced organoids were treated for 8 days with lisinopril, a frequently prescribed ACEi suitable for *in vitro* organoid experiments owing to its hydrophilic nature, lack of need for hepatic activation into active compound, and established effect of increasing ACE2 expression in animal models (67, 68). Lisinopril treatment was not found to impact organoid development, with organoids forming well-patterned nephrons with correct localization of proximal and distal tubule markers (Fig. 4A). Quantitative RT-PCR (qPCR) also revealed no significant difference in the expression of SARS-CoV-2 entry factors, including *ACE2*, across three independent experiments (Fig. 4B). These findings were further supported by similar infection of PT-enhanced organoids pre-treated with lisinopril for 48 h compared to untreated controls (Fig. 4C). Together with the entry factor expression levels, these results indicate that treatment with the ACEi lisinopril does not increase susceptibility to SARS-CoV-2 infection.

## DISCUSSION

Despite evidence for direct PT cell infection by SARS-CoV-2 (17), the exact mechanism of viral entry and pathogenesis within the kidney has remained unclear. Viral S-protein and ACE2 receptor interaction during infection have been established in numerous tissues, including kidney (15, 17, 19–22). However, viral infection of renal cell types with undetectable ACE2 has led to the suggestion of alternate receptors (15, 21).

In the current study, using an enhanced kidney organoid model with improved PT maturity (21), we provide a comprehensive overview of SARS-CoV-2 entry factor expression, confirming the PT-specific distribution of ACE2 and the absence of viral replication in PT-enhanced organoids lacking this receptor. SARS-CoV-2 was able to undergo both endosome-restricted (CTSL/CTSB) protease-mediated S protein cleavage and membrane fusion via the TMPRSS family. In the absence of specific inhibition of individual TMPRSS family members, the comprehensive transcriptional analyses performed in the current manuscript suggested a potential role for *TMPRSS10*, with PT cluster 13 (most enriched for *ACE2*, but lowly expressing *TMPRSS2*) showing the highest *TMPRSS10* levels, correlating with the detection of its corresponding protein on the apical membrane of PTs infected by SARS-CoV-2. While there is evidence that SARS-CoV-2 can utilize TMPRSS4 for entry, a role for TMPRSS10 has not previously been demonstrated (40, 69). Whether there is a preference for TMPRSS proteases over Cathepsins in renal cells remains to be seen. While viral infectivity was equally reduced with both inhibitors despite differences in PT protease expression levels, with *CTSL/CTSB* higher than all *TMPRSS* members analyzed, inhibition efficiency at the selected dosage represents a complicating factor in determining pathway preference. Nevertheless, a flexibility in protease usage, combined with viral detection in cells with undetectable ACE2 (15, 21), lends support to the hypothesis that the virus can spread from cell to cell by endosomal entry and infection of adjacent cells without ACE2 receptor involvement, as a mechanism of evading host cell immunity (70).

Furthermore, exposure of these organoids to ACEi lisinopril did not impact entry factor expression or SARS-CoV-2 infection. Although different classes of RAS inhibitors may utilize alternate modes of action, these findings support the safety of these commonly prescribed medications in the context of circulating SARS-CoV-2 [reviewed in references (2, 46)]. However, there are limitations with organoid studies, including a lack of blood supply, immune cells, and organ cross-talk. Hence, our findings cannot exclude a role for increased levels of soluble ACE2, recently speculated to bind SARS-CoV-2 in circulation and permit cell entry via ATR1 and AVPR1B receptors (71).

In summary, this study demonstrates that SARS-CoV-2 entry into renal cells is ACE2-dependent, following which the virus utilizes both TMPRSS- and CTSL/CTSB-mediated pathways. These insights broaden our understanding of infection mechanism in the context of the human kidney.

## MATERIALS AND METHODS

### Cell lines and maintenance

iPSC lines used included CRL1502.2 [derived from WS1 CRL-1502 female fibroblasts [ATCC] using episomal reprogramming methods described in reference (21), with reprogramming plasmids and mRNA detailed in references (72, 73)], PB010/MCRIi010-A (database reference; https://hpscreg.eu/cell-line/MCRIi010-A (74), and the MCRIi010-A/*ACE2* knockout iPSC line (75). iPSC lines were maintained and expanded at 37°C, 5% CO_2_, and 5% O_2_ in Essential 8 medium (Thermo Fisher Scientific, Waltham, MA) on Matrigel- (BioStrategy, Victoria, Australia) coated plates, with daily media changes and passaging every 2–3 days with EDTA in 1× PBS as described previously (76). African green monkey kidney epithelial (Vero cells, ATCC Cat. CCL-81), Vero E6 (ATCC Cat. CRL-1586), and Vero hSLAM (Merck, Cat. 04091501) cells were cultured at 37°C and 5% CO_2_. Vero and Vero E6 cell media: Minimum Essential Media (MEM) (Media Preparation Unit, Peter Doherty Institute) supplemented with 5% Fetal Bovine Serum (FBS, Bovogen, Victoria, U.S.A), 50 U/mL Penicillin and 50 µg/mL Streptomycin (PenStrep, Thermo Fisher Scientific), 2 mM GlutaMAX (Thermo Fisher Scientific), and 15 mM HEPES (Thermo Fisher Scientific). Vero hSLAM cell media: MEM supplemented with 7% FBS, PenStrep, 2 mM GlutaMAX, 15 mM HEPES, and 0.4 mg/mL G418 Sulfate (Gibco).

### Directed differentiation and PT-enhanced kidney organoid generation

For PT-enhanced kidney organoid generation, iPSCs and ESCs were seeded into laminin-coated 12-well plates at a density of 25,000 cells/well and differentiated for 13 days (including 5 days of CDBLY2 exposure) prior to manual organoid generation according to methods detailed in reference (21).

### Immunofluorescence and confocal microscopy

For immunofluorescence, organoids were fixed and stained as previously described (73) using the antibodies detailed in Table 1, diluted in 0.1% TX-100/PBS. Imaging was performed on the ZEISS LSM 780 confocal microscope (Carl Zeiss, Oberkochen, Germany) with acquisition and processing performed using ZEISS ZEN Black software (Zeiss Microscopy, Thornwood, NY) and Fiji ImageJ (77).

**TABLE 1:** Antibodies used in immunofluorescence studies

Specificity	Host species	Dilution range	Manufacturer and identifier
ACE2	Rabbit polyclonal IgG	1:300	Abcam (ab15348)
Double-stranded RNA (dsRNA)	Mouse monoclonal IgG2a, Kappa	1:100	Absolute Antibody (Ab01299-2.0)
EpCAM (Alexa488 or Alexa647 conjugate)	Mouse monoclonal IgG2a, Kappa	1:300	BioLegend (324210 and 324212)
NEPHRIN	Sheep polyclonal IgG	1:300	R&D Systems (AF4269)
Proximal tubule brush border membrane	*Lotus tetragonobulus* lectin (LTL)	1:300–1:500	Vector Laboratories (B-1325)
TMPRSS2	Mouse monoclonal IgG1	1:300	Merck (MABF2158-25UG)
TMPRSS4	Polyclonal Rabbit IgG	1:300	Thermo Fisher Scientific (PA5-999809)
TMPRSS10	Monoclonal rat IgG2A	1:300	R&D Systems (MAB2209)
SLC12A1	Rabbit polyclonal IgG	1:300–1:400	Proteintech (18970-1-AP)

### SARS-CoV-2 viruses

SARS-CoV-2 virus hCoV-19/Australia/VIC01/2020 (VIC01, GISAID ID: EPI_ISL_406844) was a kind gift obtained from the Victorian Infectious Diseases Reference Laboratory (VIDRL). icSARS-CoV-2-GFP (Wuhan/WA1) virus was a kind gift from Prof Ralph S. Baric from the Department of Microbiology and Immunology, University of North Carolina at Chapel Hill, Chapel Hill, NC, USA (78). VIC01 was propagated in Vero and Vero hSLAM cells in serum-free MEM in the presence of 1 µg/mL TPCK-Trypsin (Worthington Biochemical Corp, NJ, USA). icSARS-CoV-2-GFP was passaged in either Vero E6 cells without TPCK-Trypsin or Vero hSLAM cells with 1 µg/mL of TPCK-Trypsin. Virus stocks were stored at −80°C and titered by Median Tissue Culture Infectious Dose assay (TCID_50_) using Vero cells. All viruses were sequenced via Illumina deep sequencing. VIC01 virus stock contained three amino acid changes (R685G, A5844V, D4432A). These mutations were present in a minor fraction of the sequence reads (<10%). icSARS-CoV-2-GFP virus stock contained a single amino acid change (S6299Y) in ORF1ab and three deletions in Spike (nucleotide positions 23598–23599, 23600–23618, and 23628–23636). This included a six amino acid deletion at positions 680–685 corresponding to the furin-cleavage site. This virus stock was a mixed population with the furin-cleave site deletion present in <40% of the sequencing reads in virus passage 1.

### SARS-CoV-2 infection

PT-enhanced kidney organoids grown on Transwells were infected at day 12 of organoid culture for 3 h with 10^4^ tissue culture infectious dose (TCID_50_) of SARS-CoV-2 virus added to the apical Transwell compartment prior to media sampling every second day according to the methods outlined in (21). Media samples were subjected to viral titration by Median Tissue Culture Infectious Dose assay (TCID_50_) using Vero cells.

### Drug treatment assays

For all drug treatment assays, PT-enhanced organoids grown on Transwells were pre-treated at day 10 of organoid culture for 3 h (entry inhibitors) or 48 h (lisinopril) prior to viral infection, with 1 mL of drug-containing media added to the basolateral compartment and refreshed every second day up until the day 6 endpoint. For inhibition of proteases involved in viral infection, organoids were treated with either 10 µM of Camostat mesylate (Sigma Aldrich, MO, USA, cat. SML0057), 10 µM of E64d (Selleck Chem, TX, USA, cat. S7393), Camostat + E64 d combined (10 µM each), or an equivalent concentration of the drug reconstitution reagent, DMSO (Sigma Aldrich). For investigating to influence of ACEi on infectivity, organoids were treated with 1 mM lisinopril (Sigma Aldrich, cat. L6292-100MG) or remained untreated. Infectivity was assessed as outlined in “SARS-CoV-2 infection” above.

### Real-time quantitative reverse transcription PCR

RNA extraction, cDNA synthesis, and quantitative RT-PCR (qRT-PCR) were performed using the Bioline Isolate II Mini/Micro RNA Extraction Kit, SensiFAST cDNA Synthesis Kit, and the SensiFAST SYBR Lo-ROX Kit (Bioline, NSW, Australia) according to the manufacturer’s instructions. Triplicate technical replicates were performed for each reaction using the primer pairs detailed in Table 2. RT-PCR data were collected using the Applied Biosystems 7500 Sequence Detection Software (version 1.5.1) installed on the Applied Biosystems 7500 Real Time PCR System. Data were graphed and analyzed in Prism 9 (GraphPad).

**TABLE 2:** Forward and reverse primers used for qRT-PCR

Gene	Forward primer (5′−3′)	Reverse primer (5′−3′)
*ACE*	CAACCTGCATGCCTACGTG	GCGTCCAGCCCTGCTTTA
*ACE2*	GGGATCAGAGATCGGAAGAAGAAA	AGGAGGTCTGAACATCATCAGTG
*CTSL*	GACGCGGTCGAGTAGGTTTT	GGCAATTCCCAGGCAAAAGG
*CTSB*	GCTTCGATGCACGGGAACAATG	CATTGGTGTGGATGCAGATCCG
*GAPDH*	CTCTCTGCTCCTCCTGTTCGA	TGAGCGATGTGGCTCGGCT

### Single cell RNA (scRNAseq) analyses

ScRNAseq analyses were performed on our existing published single cell data set of PT-enhanced organoids (GEO accession: GSE184928) (21). This data set consisted of >11,000 cells from four hashtag oligo barcoded replicates. Library demultiplexing in CellRanger, normalization, and marker analysis in Seurat (3.1.4) were performed as described previously (21). Code for the scRNAseq analyses in the current study is available in the Kidney Regeneration Github repository (https://github.com/KidneyRegeneration/Vanslambrouck2023).

## Data Availability

ScRNAseq analyses in the current study were performed on our existing published single cell dataset of PT-enhanced organoids (GEO accession: GSE184928). Code for the scRNAseq analyses in the current study is available in the Kidney Regeneration Github repository (https://github.com/KidneyRegeneration/Vanslambrouck2023).

## SUPPLEMENTAL MATERIAL

Figure S1 jvi.01802-23-s0001.tif PT-enhanced organoid infectivity and inhibition.

Supplemental legend jvi.01802-23-s0002.docx Legend for Fig. S1.

ASM does not own the copyrights to Supplemental Material that may be linked to, or accessed through, an article. The authors have granted ASM a non-exclusive, world-wide license to publish the Supplemental Material files. Please contact the corresponding author directly for reuse.
