# Supplementary figures and images for: Kidney organoids reveal redundancy in viral entry pathways during ACE2-dependent SARS-CoV-2 infection
Source: J Virol. 2024 Feb 9;98(3):e01802-23. doi: 10.1128/jvi.01802-23 (PMC10949421; doi:10.1128/jvi.01802-23)

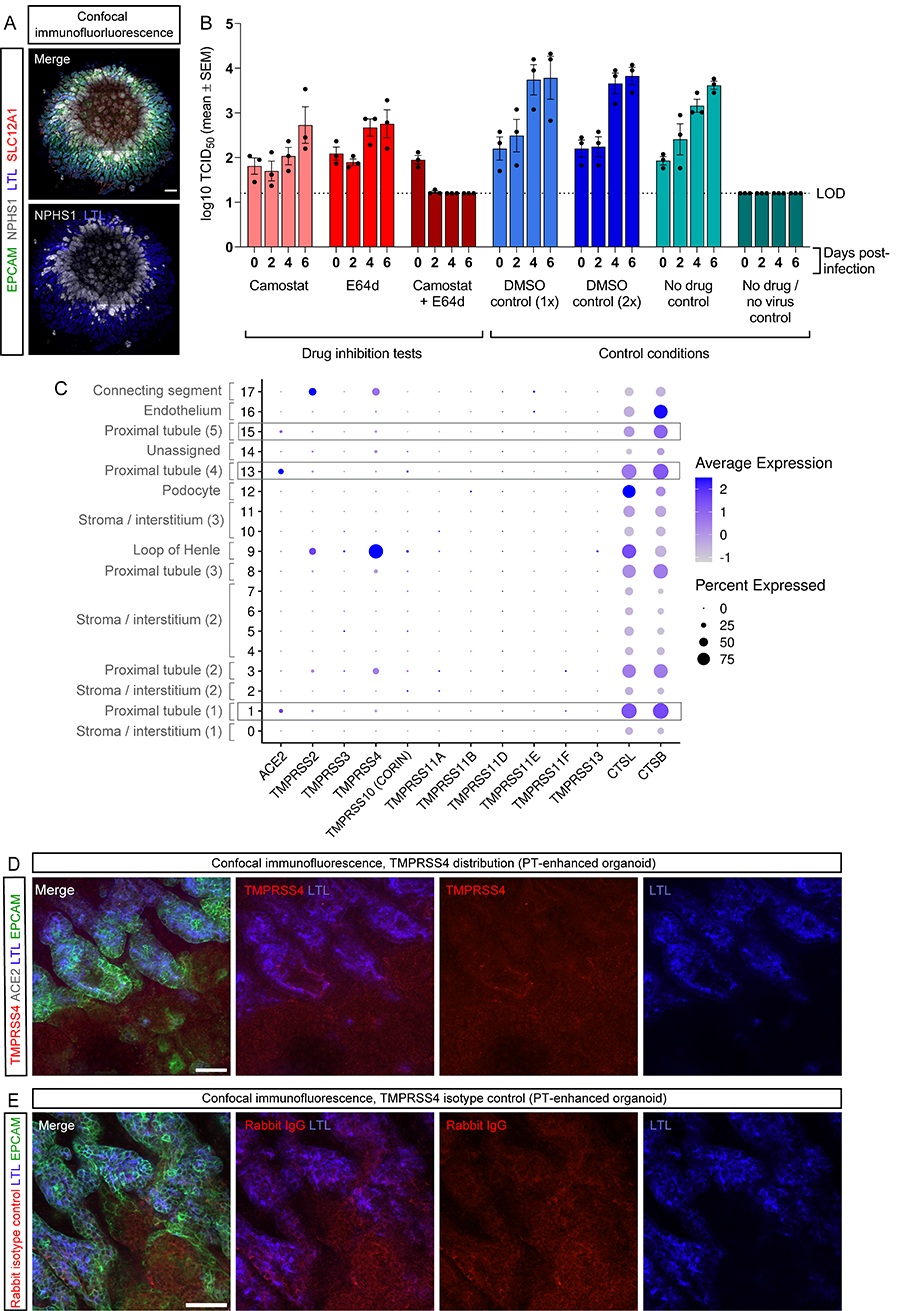

Supplement: Figure S1 — PT-enhanced organoid infectivity and inhibition. [file jvi.01802-23-s0001.tif]
