# Supplementary material for: Kidney organoids reveal redundancy in viral entry pathways during ACE2-dependent SARS-CoV-2 infection
Source: J Virol. 2024 Feb 9;98(3):e01802-23. doi: 10.1128/jvi.01802-23 (PMC10949421; doi:10.1128/jvi.01802-23)
Supplement: Supplemental legend — Legend for Fig. S1. [file jvi.01802-23-s0002.docx]

**Kidney organoids reveal redundancy in viral entry pathways during ACE2-dependent SARS-CoV-2 infection**

Jessica M. Vanslambrouck^1,2*^, Jessica A. Neil^3*^, Rajeev Rudraraju^3*^, Sophia Mah^1^, Ker Sin Tan^1^, Thomas A Forbes^1,2,4^, Katerina Karavendzas^1^, David A Elliott^1,2,5^, Enzo R Porrello^1,6,7^, Kanta Subbarao^3,8^^, Melissa H. Little^1,2,9^#^.

1. The Novo Nordisk Foundation Centre for Stem Cell Medicine (reNEW), Murdoch Children's Research Institute, Parkville, Melbourne, Australia.
2. Department of Paediatrics, Faculty of Medicine, Dentistry and Health Sciences, The University of Melbourne, Parkville, Melbourne, Australia
3. Department of Microbiology and Immunology, The Peter Doherty Institute for Infection and Immunity, The University of Melbourne, Australia.
4. Department of Nephrology, Royal Children's Hospital, Parkville, Melbourne, Australia.
5. Australia Regenerative Medicine Institute, Monash University, Clayton, Victoria, Australia.
6. Melbourne Centre for Cardiovascular Genomics and Regenerative Medicine, The Royal Children’s Hospital, Melbourne, Australia.
7. Department of Anatomy and Physiology, School of Biomedical Sciences, The University of Melbourne, Parkville, Melbourne, Australia.
8. The WHO Collaborating Centre for Reference and Research on Influenza, The Peter Doherty Institute for Infection and Immunity, Melbourne, Australia.
9. Novo Nordisk Foundation Centre for Stem Cell Medicine (reNEW), Faculty of Health and Medical Sciences, University of Copenhagen, Denmark

***Equal first author contribution**

**^Equal last author contribution**

**^#^Author for correspondence:** M.H.L.: +61 3 9936 6206; [melissa.little@mcri.edu.au](mailto:melissa.little@mcri.edu.au)

**Running title:** SARS-CoV-2 renal entry mechanism

**Supplementary Figure 1 Legend**

**Supplementary Figure 1:** **PT-enhanced organoid infectivity and inhibition. A.** Confocal immunofluorescence of a representative PT-enhanced kidney organoid at 14 days of organoid culture. Images (top and bottom) depict merged and separated channels, showing nephron epithelium (EPCAM; green), podocytes of glomeruli (NPHS1; grey), proximal tubules (LTL; blue), and loop of Henle (SLC12A1; red). Scale bar represents 200 µm. **B.** Bar graph depicting the viral titres (log10 TCID_50_/mL) of culture media sampled from PT-enhanced organoids at 0 - 6 days post-infection, treated with either protease inhibitors (Camostat and E64d, alone or in combination; dark/light red bars), drug reconstitution reagent (DMSO controls; dark/light blue bars), or remaining untreated (no drug controls; light/dark green bars). PT-enhanced organoids were infected with WA1 SARS-CoV-2 (icSARS-CoV-2-GFP). LOD and dotted line represents lower limit of detection. Error bars represent SEM from 3 independent experiment, with 3 (drug inhibition tests and no drug controls) or 2 (DMSO controls) biological replicates per timepoint. **C.** scRNAseq DotPlot of PT-enhanced organoids (day 14 of organoid culture) depicting the expression of *ACE2*, *TMPRSS* family and *CTS* family genes within all clusters. Clusters displaying the highest *ACE2* expression are outlined with grey boxes. Dot size represents the percentage of cells expressing a gene within each cluster, while shade intensity correlates with gene expression level. **D-E.** Confocal immunofluorescence of TMPRSS4 (C, red) and corresponding rabbit isotype control (D, red), co-stained with markers of proximal tubule (LTL, blue) and nephron epithelium (EPCAM, green). Scale bars represent 50 µm.
